# EXERCISE TOLERANCE, PULMONARY FUNCTION, RESPIRATORY MUSCLE STRENGTH, AND QUALITY OF LIFE IN CHILDREN AND ADOLESCENTS WITH RHEUMATIC HEART DISEASE

**DOI:** 10.1590/1984-0462/;2018;36;2;00012

**Published:** 2018-03-29

**Authors:** Andressa Lais Salvador de Melo, Yasmin França Bezerra de Lira, Luziene Alencar Bonates Lima, Fabiana Cavalcanti Vieira, Alexandre Simões Dias, Lívia Barboza de Andrade

**Affiliations:** a Instituto de Medicina Integral Prof. Fernando Figueira, Recife, PE, Brasil.; b Faculdade Pernambucana de Saúde, Recife, PE, Brasil.; c Universidade Federal do Rio Grande do Sul, Porto Alegre, RS, Brasil.

**Keywords:** Rheumatic heart disease, Child, Adolescent, Spirometry, Exercise tolerance, Quality of life, Cardiopatia reumática, Criança, Adolescente, Espirometria, Tolerância ao exercício, Qualidade de vida

## Abstract

**Objective:**

Despite the high prevalence of rheumatic heart disease in Brazil, the occurrence of functional impairment in children and adolescents with rheumatic heart disease is not clear. The aim of this study was to evaluate exercise tolerance, respiratory muscle strength, lung function, and quality of life of children and adolescents with rheumatic heart disease.

**Methods:**

Cross-sectional study, conducted from August to December 2014 with children and adolescents with rheumatic heart disease aged 8 to 16 years. The participants, after completing the socioeconomic, clinical, and quality of life questionnaires were tested by spirometry, manovacuometry and in a 6-minute walk test. The variables and their reference values were compared using the paired Student’s t-test. Comparisons between predicted and observed walking distance were done also by Student’s t-test, consdiering the categorization of the participants. Correlations between these differences and quantitative variables were assessed by Pearson’s coefficient, being significant *p*<0.05.

**Results:**

All 56 participants had a walked distance lower than predicted (*p*<0.001). The differences between predicted and observed distances were positively correlated with the baseline heart rate (r=0.3545; *p*=0.007). Expiratory muscle strength was also lower than the predicted values (*p*<0,001). Regarding quality of life assessment, the mean scores were 70, 77 and 67% for general, physical, and psychosocial aspects, respectively.

**Conclusions:**

Children and adolescents with rheumatic heart disease have reduced exercise tolerance, which is related to their higher baseline heart rate; they also show impaired expiratory strength and quality of life.

## RESUMO

**Objetivo:**

Apesar da alta prevalência de cardiopatia reumática no nosso país, a ocorrência de prejuízos funcionais em crianças e adolescentes com cardiopatia reumática não está esclarecida. Este estudo visou avaliar tolerância ao exercício, força muscular respiratória, função pulmonar e qualidade de vida de crianças e adolescentes com cardiopatia reumática.

**Métodos:**

Estudo transversal, realizado de agosto a dezembro de 2014, com portadores de cardiopatia reumática de 8 a 16 anos de idade. Os participantes, após preenchimento dos questionários socioeconômico, clínico e de qualidade de vida, foram submetidos a espirometria, manovacuometria e teste de caminhada de seis minutos. As variáveis e seus valores de referência foram comparados pelo teste *t* de Student pareado. Para comparar as diferenças entre as distâncias percorridas prevista e observada, considerando-se as categorizações dos participantes, foi utilizado o teste *t* de Student. Correlações entre essas diferenças e as variáveis quantitativas foram feitas pelo coeficiente de Pearson, sendo significante *p*<0,05.

**Resultados:**

Os 56 participantes obtiveram distância percorrida inferior à prevista (*p*<0,001). As diferenças entre as distâncias prevista e observada mostraram correlação positiva com a frequência cardíaca basal (r=0,3545; *p*=0,007). A força muscular expiratória também foi inferior à prevista (*p*<0,001). A qualidade de vida foi de 70; 77 e 67%, respectivamente, nos domínios geral, físico e psicossocial.

**Conclusões:**

Crianças e adolescentes com cardiopatia reumática apresentam tolerância reduzida ao exercício, a qual está relacionada com uma maior frequência cardíaca basal; eles também demonstram prejuízo na força expiratória e na qualidade de vida.

## INTRODUCTION

Children and adolescents with acquired heart diseases may be functionally impaired by the disorder itself, associated respiratory changes, life habits, and other factors that lead to a poor quality of life.^1^ ^,^ ^2^

Chronic rheumatic valvulopathy is the leading form of acquired heart disease among children, adolescents, and young adults, with high potential for morbidity and mortality in this population. Developed countries have already been able to significantly reduce cases,^3^ but in some parts of the world such as Brazil and other Latin American countries, the incidence remains high, about three cases per thousand inhabitants^4^, due to factors such as poverty, low per capita income, bad housing conditions, and poor access to health services.^5^ In Pernambuco, severe forms of chronic rheumatic heart disease have been reported, mainly due to lack of adequate treatment for patients with streptococcal pharyngotonsillitis and failure of secondary prophylaxis in patients with cardiac sequelae.^6^

Few studies with children and adolescents presenting congenital heart disease correlate aspects of pulmonary function with reduced ability to exercise,^7^ ^,^ ^8^ but these outcomes can not be extended to children and adolescents with rheumatic heart disease. The literature also lacks information about the quality of life of this population. It is suggested that children with both acquired or congenital cardiovascular diseases have a perception of lower quality of life compared to healthy children, both in physical and psychosocial aspects.^9^

Considering the high prevalence of rheumatic heart disease in some regions of the country, more attention should be given to the evaluation of the disease’s impact measures, such as impairment of daily activities, functionality, quality of life, and health conditions.^10^ Therefore, the purpose of this study was to evaluate exercise tolerance, pulmonary function, respiratory muscle strength, and quality of life of children and adolescents with rheumatic heart disease.

## METHOD

A cross-sectional study was carried out involving children and adolescents with rheumatic heart disease recruited from August through December 2014 at the Pediatric Cardiology Outpatient Clinic of *Instituto de Medicina Integral Professor Fernando Figueira* (IMIP). This is a philanthropic school hospital, reference in the north/northeast regions of Brazil for children’s health, whose demand comes from the Brazilian Public Health System (SUS) and which holds a cardiology outpatient clinic that assists 40 patients per month, on average, for research on and follow-up of rheumatic fever.

The sample was consecutively selected for convenience during the study and met the following inclusion criteria: age between 8 and 16 years, more than one year since the last episode of rheumatic fever and three months since the last surgery. Patients with congestive heart failure degrees III or IV were excluded, as well as those with unstable angina or history of acute myocardial infarction in the previous month, comorbidities such as chronic kidney disease and any pneumopathy, gestation, difficulty understanding the questionnaires and tests; changes in the motor system and initial physiological parameters that contraindicated the six-minute walk test (6MWT), including heart rate (HR) >120 bpm, systolic blood pressure (SBP) >180 mmHg, and diastolic blood pressure (DBP) >100 mmHg.^11^

The diagnosis of rheumatic fever established by the pediatric cardiology service had Jones’ criteria modified as follows.^12^ Cardiopathy is identified by echocardiography and the Valve Guidelines are followed for severity of valve dysfunction.^13^

The research was previously approved by the Research Ethics Committee of the institution, in accordance with Resolution 466/12 of the National Health Council, under CAAE approval number 27915614.0.0000.5201. Participants signed an informed consent form, as did their parents/caregivers.

Data collection and assessment were standardized and performed by two properly trained members of the research group. Collection was started with the completion of a form regarding socioeconomic (participant’s and parents/caregivers’ education, and family income) and clinical information, including age, sex, weight, height, body mass index (BMI), valve lesion severity, functional class by the New York Heart Association (NYHA),^14^ and usual physical activity level). The latter was measured by a score adapted from the Habitual Level of Physical Activity (HLPA) questionnaire,^15^ in which the following gradations were established: 1 (sedentary), 2 (regular activity up to two hours per week) and 3 (competitive/organized activity for at least three hours per week). Weight and height were obtained by the use of an anthropometric scale (Filizola^®^, São Paulo, SP, Brazil), and subjects were sorted by BMI as: low-weight, eutrophic, overweight, and obese.^16^ Evaluations were made in the following sequence:

1. quality of life;
2. respiratory function;
3. respiratory muscle strength;
4. 6MWT.

Quality of life was evaluated by the Pediatric Quality of Life Inventory (PedsQL), validated for Brazil by Klatchoian et al.^17^ and originally conceived by researcher James W. Varni, who authorized its use for the purposes of this study. The instrument is customized for each age group. It contains 23items organized in four domains: physical (eight items), emotional (five items), social (five items) and school (five items). For each item, the participant had to choose one from five options (never, almost never, sometimes, often, or almost always). For each response, a score from zero to four would be assigned, then transferred to a range from zero to 100 and converted into percentage. The closer to 100%, the better the quality of life.

Pulmonary function tests were performed using a One-Flow portable spirometer (Clement Clark^®^, England), resulting in forced vital capacity (FVC), forced expiratory volume in the first second (FEV_1_), and FEV_1_/FVC ratio of maximum inspiration followed by forced expiration, as recommended by the American Thoracic Society (ATS).^18^ Predicted values were obtained from the online calculator by the Global Lung Function Initiative.^19^

Respiratory muscle strength was measured by an analogical manovacuometer (Comercial Médica^®^, São Paulo, SP, Brazil). All tests were performed as recommended by ATS.^20^ Maximum inspiratory pressure (MIP) was measured in a forced and sustained inspiratory maneuver, starting at maximum expiration. The maximum expiratory pressure (MEP) was evaluated by a forced and sustained expiratory maneuver, starting at maximum inspiration. Reference values were obtained as proposed by the equations by Lanza et al.^21^

Exercise tolerance was assessed by the 6MWT, according to ATS standards.^11^ Before and after the test HR, RF, peripheral oxygen saturation (SpO_2_), blood pressure (BP), dyspnea sensation, and lower limb fatigue sensation were measured. HR and SpO_2_ were collected by a pulse oximeter (EMAI^®^, model OXP-10, São Paulo, SP, Brazil). RF was evaluated by inspection of thoracoabdominal movements within one minute and marked by a digital timer (Junsd^®^ JS-307, Guangdong, China). BP was assessed by indirect method of auscultation with a sphygmomanometer (Tycos^®^ Welch Allyn DS58MC, New York, USA) and a stethoscope (Littmann^®^ Classic II, Weymouth Dorset, UK). Dyspnea and lower limb fatigue sensation were assessed by the modified Borg scale,^22^ from zero to ten, where zero means no symptoms and ten stands for the worst sensation of dyspnea or fatigue. The 6MWT was performed in a 20-meter-long corridor delimited by two signaling cones that also served as markers for both ends of the path, as suggested by Aquino et al.^23^

Participants were each instructed to move as fast as possible, but not run, for six minutes from one cone to the other, being bypassed every time they were reached. At the end of the 6MWT, the distance covered and the difference between predicted (Dp) and observed (Do) values were verified. To minimize the learning effect and reduce possible measurement errors, each participant performed the test twice with a ten-minute interval, and only the measurements of the second attempt were recorded for analysis. From these values, ​the difference of means between distance covered by the patient and the distance predicted for the 6MWT was obtained. Predicted values ​​were calculated using the equation by Priesnitz et al.^24^ for participants aged ≤12 years and the equation by Iwama et al.^25^ for those aged 13+ years.

The data were input to a spreadsheet of Excel 2013 (Microsoft Office, Las Vegas, USA) and processed in the statistical program Stata version 12.0 (StataCorp LLC, Texas, USA). The descriptive information was expressed in absolute numbers and proportions, means and standard deviations (SD) when pertinent. Analytical data were expressed as mean and standard deviation. The variables DO, FVC, FEV_1_, FEV_1_/FVC, maximum inspiratory pressure (MIP), maximum expiratory pressure (MEP) and respective reference values ​​were compared by the paired Student’s t test, for the samples were not independent. To compare means of differences between distance covered and predicted distance, the Student’s t test was used while considering participants’ categorization. Finally, the Pearson’s correlation coefficient was used to test correlations between Dp and Do and the variables FVC, FEV_1_, FEV_1_/FVC, MIP, MEP, quality of life scores, and cardiorespiratory parameters measured upon the 6MWT (HR, RR, SpO_2_, level of dyspnea and lower limbs fatigue sensation), age and BMI. The statistical significance level adopted for all analyses was 0.05.

## RESULTS

A total of 56 subjects participated in the study, 30 (53.6%) of them being males, with mean age 12.9±2.1 years. They all had insufficiency of one or more heart valves (78.5% had mild or moderate dysfunction) and were classified as Functional Class I, according to NYHA criteria. Mean QoL scores for the physical domain was 76.8±2.0% and for the psychosocial domain - which encompasses emotional, social, and school spheres -, 67.1±1.8%; the overall result was 70.53%. Table 1 shows all other categorical characteristics of the sample.

**Table 1:** Characteristics of patients (n=56) with rheumatic heart disease.

	n	%
Male gender	30	53.6
BMI		
Low-weight	5	8.9
Eutrophic	34	60.7
Overweight	9	16.1
Obesity	8	14.3
Monthly family income		
2-4 minimum wages	2	3.6
Less than 2 minimum wages	54	96.4
Education of head of family ≤8 years	43	76.8
Inadequate education of participant	24	42.8
HLPA		
Sedentarism	26	46.4
Up to 2h of physical activity weekly	14	25.0
Competitive/organized physical activity ≥3h weekly	16	28.6
Severity of valve lesion		
Mild	26	46.4
Moderate	18	32.2
Severe	12	21.4

BMI: body mass index; HLPA: Habitual Level of Physical Activity

The mean values for the variables evaluated before and after the 6MWT were: HR between 79±12 and 117±23 beats per minute (bpm), RR between 15.7±2.7 and 24.0±3.5 respiratory incursions per minute (ripm), pulse oxygen saturation between 98.2±1.3 and 97.8±2.4%, SBP between 119±13 and 131±16 mmHg and DBP between 73.6±8.5 and 80±10 mmHg.

Table 2 shows a comparison between mean observed and predicted values of respiratory muscle strength, pulmonary function, and exercise tolerance, with expiratory muscle strength and exercise tolerance significantly lower than predicted.

**Table 2:** Comparison between observed and predicted values of respiratory muscle strength, lung function, and exercise tolerance in children with rheumatic heart disease, Recife, 2014.

	Observed	Predicted	p-value
MIP (cmH_2_0)	99.50	101.30	0.540
MEP (cmH_2_0)	91.00	102.50	<0.001
FVC (L)	3.02	3.16	0.360
FEV_1_ (L)	2.66	2.76	0.380
FEV_1_/FVC	0.90	0.88	0.050
Distance covered (m)	516.20	625.00	<0.001

Values expressed as mean; MIP: maximum inspiratory pressure; MEP: maximum expiratory pressure; FVC: forced vital capacity; FEV_1:_ forced expiratory volume in the first second.

Table 3 presents comparisons of differences between Dp-Do, taking the categorization of participants into account as per baseline characteristics. No statistical difference was found between the data compared.

**Table 3:** Comparison of differences between predicted and observed distance covered in the six-minute walk test, according to individuals’ characteristics.

	n	Dp-Do	SD	p-value
Gender				
Male	30	120.92	81.68	0.2431
Female	26	98.44	55.08	
Valve lesion				
Mild	26	103.64	71.49	0.5087
Moderate	18	118.24	73.53	0.6003
Important	12	105.53	59.98	0.9330
Physical activities				
HLPA 1 and 2	40	127.29	75.14	0.0589
HLPA 3	16	92.83	59.90	

Dp-Do: predicted minus observed distance covered (mean); SD: standard deviation; HLPA 1: sedentary; HLPA 2: practicing physical activity up to 2h/weekly; HLPA 3: practicing organized/competitive physical activity at least 3h/weekly.

Finally, Table 4 shows correlations between Dp-Do and other quantitative variables, noting that only baseline heart rate was shown correlated with Dp-Do.

**Table 4:** Correlations of mean of differences between predicted and observed distances covered with some variables.

	r	p-valor
MIP	-0.136	0.31
MEP	0.090	0.50
FVC	0.136	0.31
FEV_1_	0.183	0.18
FEV_1_/FVC	-0.042	0.76
Physical QoL	-0.158	0.24
Psychosocial QoL	0.015	0.91
Overall QoL	-0.058	0.67
∆HR	-0.203	0.13
Initial HR	0.354	0.007*
Final HR	-0.008	0.95
∆MBP	-0.052	0.70
∆Borg (dyspnea)	0.082	0.54
∆Borg (LL fatigue)	-0.122	0.36
∆RR	-0.230	0.08
BMI	-0.123	0.36
Age	0.240	0.07

MIP: maximum inspiratory pressure; MEP: maximum expiratory pressure; FVC: forced vital capacity; FEV_1:_ forced expiratory volume in the first second; QoL: quality of life; HR: heart rate; ∆MBP: final minus initial mean blood pressure; ∆HR: final minus initial heart rate; ∆Borg: Final minus initial Borg; ∆RR: final minus initial respiratory rate; BMI: body mass index; *p<0.05; Pearson’s correlation coefficient.

## DISCUSSION

Deficit in exercise tolerance, represented by the significantly lower distance covered then predicted on the 6MWT for children and adolescents with rheumatic heart diseases, even if they were clinically stable and sorted as Functional Class I as per NYHA, was the main finding of this study. In addition, a poor quality of life was detected in this population.

Few studies have addressed exercise tolerance by children and adolescents with heart diseases; those addressing the topic have employed different tests and none has evaluated individuals with rheumatic heart disease. The other study that used the 6MWT in children and adolescents with corrected congenital heart diseases, likewise this paper, reported a significant deficit in distance covered compared to predicted values.^26^

In order to correlate exercise tolerance with the other variables evaluated, Dp-Do was used, representing the performance in 6MWT, since the isolated Do value varies greatly according to one’s physical characteristics. Positive correlation was found between Dp-Do and HR measured before the test, meaning that the higher the participant’s baseline HR, the lower their exercise tolerance. This finding may be justified by the influence of heart function in this context, but no specific cardiac evaluation was performed at the time of the study. In addition, when comparing 6MWT scores between participants with prior diagnosis of mild, moderate, and severe valve dysfunction, no significant difference was found as to tolerance. According to ATS,^11^ the 6MWT evaluates the response of several systems involved in exercise, including respiratory, cardiovascular, systemic and peripheral circulation, neuromuscular units, and muscle metabolism, which suggests an overall assessment of exercise tolerance.

Respiratory system function was assessed by respiratory muscle strength and lung function. No significant changes were identified between individuals of the sample. The only study in the literature addressing lung function of children and adolescents with rheumatic heart disease reported spirometry values below expectations, but subjects had been evaluated in pre- and postoperative periods of heart surgery, which may have affected the results negatively.^27^

Regarding the response of respiratory muscle strength, participants showed inspiratory strength within the expected and expiratory strength below the expected. Feltez et al.,^26^ in addition to finding a deficit in 6MWT, reported children and adolescents with congenital heart disease with expiratory muscle strength lower than expected, which gives support to our findings. Although not measured, MEP values do not appear to have a great impact on the respiratory system function in a moment of clinical stability, but in a postoperative period, it is likely to cause impairments such as cough inefficiency and consequent airway protection failure.

Changes in expiratory muscle strength may reflect difficulties in airflow generation due to impaired lung function, which was not confirmed in this study. Another hypothesis is skeletal muscle function impairment caused by either physical inactivity and/or changes in muscle metabolism, which has been previously demonstrated in adults with acquired heart diseases, and in children and adolescents with congenital heart diseases.^27^ ^,^ ^28^ As there is evidence that hypotrophy and muscle weakness are related to reduced exercise tolerance,^29^ ^,^ ^30^ this hypothesis may explain the results obtained by both the 6MWT and the manovacuometry, besides supporting the finding of 46% of sedentary individuals in the sample.

In this study, muscle fatigue evaluation was performed subjectively by Borg scale,^22^ but no correlation with the results of 6MWT was found, likewise with respiratory data and variables other than baseline HR.

As to lifestyle, more active individuals, who practiced at least three hours of organized and/or competitive physical activity weekly, covered distances closer to the expected when compared to others, but this study was not intended to such comparison.

O’Byrne et al.^31^ assessed heart function, habitual physical activity level, and exercise tolerance in 208 children with congenital heart disease, concluding that exercise tolerance is more correlated to physical activity level than to heart function. Sampaio et al.^32^ studied the effects of enalapril maleate therapy on heart function and exercise tolerance of subjects with asymptomatic or mild symptomatic mitral regurgitation secondary to valve prolapse or rheumatic heart disease, and reported that the treatment was able to improve heart function, but not to alter patients’ exercise tolerance; that is, it appears that heart function alone does not determine one’s ability to tolerate physical exertion.

In addition to pathophysiological and functional issues, the increase in life expectancy of this population turns their quality of life into an important health concern. According to Ferguson and Kovacs,^33^ some aspects contribute to a poor quality of life, including low social support, emotional instability, poor health, and low exercise tolerance. Quality of life scores were around 70%, corroborating the findings by Moraes et al.,^34^ who evaluated the same population profile aiming to validate the heart module of PedsQL for Brazil. Although there are no cutoff points described in the literature, these authors considered that these levels correspond to a lower quality of life, as per the study by Klatchoian et al.,^17^ who, found a score of 72% for children with chronic rheumatic diseases compared to healthy ones, with scores of 88%, while validating the general PedsQL for Brazil - a significant difference. Multiple factors interfere with quality of life of children and adolescents with congenital and acquired heart diseases, including socioeconomic issues.^35^

The sample studied had significant rates of school delay and low parental schooling, besides being formed by individuals with low financial resources, which may have contributed to their quality of life results. Physical function also influenced quality of life; like in our study, Moraes et al.^34^ reported children and adolescents with congenital or acquired heart disease presenting low scores on the physical domain of quality of life questionnaires; however, no study has compared these responses by objective physical assessments such as 6MWT. Gratz et al.^36^ used a quality of life questionnaire and noted that adolescents and adults with congenital heart diseases and lower physical function scores had reduced exercise tolerance. In our study, even though a statistically significant impairment in exercise tolerance was found, it was not correlated with quality of life.

In our investigation, a difference of 108 meters between distance covered and predicted distance was found, which is statistically significant. However, one can not state whether this difference is clinically significant, once the lack of studies results in cut-off points not yet established.

Some limitations of our study should be considered, though. First, the means to assess the level of physical activity was subjective, through self-report by children and adolescents about their habitual activities over the month prior to the intervention. There was not a control group to compare variables with, as well as regression equations were not used for Brazilian children and adolescents, as done in other studies;^37^ ^,^ ^38^ however, this population may present anthropometric variability due to regional differences, with potential interference in performance of some measures. In order to minimize this bias, a regression equation was used to analyze muscle strength in children from different regions of Brazil.^21^ The number of participants was sufficient to demonstrate a difference between distances covered and predicted distances, as well as a correlation between these distances and baseline HR, but not to show differences in the other analyses.

Despite the limitations, this research outlined the profile of a population that had not been studied before, but whose life expectancy had been increasing, and raised relevant information that could serve as an incentive for further studies on the physical, functional and quality of life features of children and adolescents with acquired heart disease. Conclusion is that the majority of children and adolescents with rheumatic heart disease presents reduced exercise tolerance; amongst participants, higher basal HRs were related to worse results. In addition, lower expiratory muscle strength and reduced quality of life were detected in patients with rheumatic heart disease.
